# Pre-Amyloidosis Red-Flag Clinical Diagnoses in Light Chain (AL) Versus Age-Related Transthyretin (ATTRwt) Amyloidosis: Electronic Health Record–Based Descriptive Study

**DOI:** 10.2196/85803

**Published:** 2026-06-22

**Authors:** Noel Estrada-Merly, Liliana E Pezzin, Rodney Sparapani, Anita D'Souza

**Affiliations:** 1 Department of Medicine, Division of Hematology/Oncology, Medical College of Wisconsin, 9200 W Wisconsin Ave, Office #C4204, Milwaukee, WI, 53226, United States, 1 414 805 0637; 2 Division of Epidemiology and Social Sciences, Institute of Health and Humanity, Medical College of Wisconsin, Milwaukee, WI, United States; 3 Division of Biostatistics, Data Science Institute, Medical College of Wisconsin, Milwaukee, WI, United States

**Keywords:** AL amyloidosis, wild type ATTR, red-flag diagnoses, electronic health records algorithms, early detection, electronic health records, EHR, transthyretin

## Abstract

This electronic health record–based study highlights distinct diagnostic patterns preceding a diagnosis of light chain (AL) and wild-type transthyretin (ATTRwt) amyloidosis; AL amyloidosis was more frequently preceded by renal, gastrointestinal, neurologic, and clonal plasma cell disorders, whereas ATTRwt amyloidosis was more commonly associated with cardiac manifestations and carpal tunnel syndrome.

## Introduction

Amyloidosis represents diseases caused by misfolded protein deposits with multiorgan involvement. The two main systemic types are light chain amyloidosis (AL), caused by a disordered plasma cell clone, and age-related amyloidosis, also known as wild-type transthyretin amyloidosis (ATTRwt), from misfolded transthyretin protein [1]. Diagnosis is often delayed due to slow, heterogeneous presentations, leading patients through multiple specialists. Older adults and Black individuals are disproportionately affected by underdiagnosis and delays [2-5]. Although Food and Drug Administration–approved therapies exist, neither condition is curable; early AL diagnosis improves outcomes, and timely ATTRwt recognition enhances treatment access [3,6]. Common AL amyloidosis “red flag” clinical diagnoses include dyspnea, fatigue, neuropathy, renal disease, heart failure, and edema, symptoms that are often nonspecific [2,7,8]. ATTRwt often presents with cardiac, musculoskeletal, and carpal tunnel syndrome symptoms [9]. We compared red-flag claims preceding diagnosis of AL or ATTRwt amyloidosis to describe differences in symptom recognition prior to diagnosis between the two diseases.

## Methods

### Study Design

Data were collected using the TriNetX real-time platform, which included deidentified patient-level electronic health record (EHR) data from 77 US health care organizations. The AL amyloidosis cohort included adults ≥18 years old with ≥2 *International Classification of Diseases, 10th Revision, Clinical Modification* (*ICD-10-CM*) codes (E85.81, E85.4, E85.89, E85.9) [5] between January 1, 2017, and December 31, 2023, and treated with plasma cell–directed chemotherapy; those receiving tafamidis were excluded. The ATTRwt cohort included patients with ≥2 occurrences of E85.82 in the same period and tafamidis treatment. Those receiving chemotherapy were excluded. Prespecified red-flag diagnoses were identified using *ICD-CM-9/10* codes (Table 1).

**Table 1.** Comparison of red-flag diagnoses at any time prior to the diagnosis of wild-type transthyretin amyloidosis (ATTRwt) or light chain amyloidosis (AL) before and after propensity score matching.

	Any time prior to diagnosis					
	Before propensity score matching			Propensity score–matched^a^		
	ATTRwt (N=2614), n (%)	AL (N=12,090), n (%)	*P* value	ATTRwt (N=2503), n (%)	AL (N=2503), n (%)	*P* value
Precursor diagnoses						
Clonal						
Multiple myeloma	59 (2)	4018 (33)	<.001	53 (2)	720 (29)	<.001
Monoclonal gammopathy	232 (9)	2596 (22)	<.001	220 (9)	550 (22)	<.001
Cardiac						
Heart failure	1872 (72)	4787 (40)	<.001	1795 (72)	1114 (45)	<.001
Cardiomyopathy	1234 (47)	2888 (24)	<.001	1190 (48)	605 (24)	<.001
Atrial fibrillation and flutter	1445 (55)	3182 (26)	<.001	1388 (56)	920 (37)	<.001
Cardiomegaly	1191 (46)	3333 (28)	<.001	1143 (46)	757 (30)	<.001
Other cardiac arrhythmias	1056 (40)	3434 (28)	<.001	1017 (42)	885 (35)	<.001
Renal						
Proteinuria	164 (6)	2320 (19)	<.001	161 (6)	422 (17)	<.001
Chronic kidney disease	961 (37)	4820 (40)	.003	914 (37)	1087 (43)	<.001
Nephrotic syndrome	<10 (<1)	860 (7)	<.001	<10 (<1)	141 (6)	<.001
Gastrointestinal/hepatic						
Constipation	463 (18)	2972 (25)	<.001	444 (18)	687 (27)	<.001
Diarrhea	277 (11)	2638 (22)	<.001	270 (11)	485 (19)	<.001
Nausea and vomiting	320 (12)	3028 (25)	<.001	304 (12)	508 (20)	<.001
Dysphagia	269 (10)	1964 (16)	<.001	258 (10)	460 (18)	<.001
Abdominal and pelvic pain	641 (25)	4110 (34)	<.001	616 (25)	818 (33)	<.001
Other diseases of liver	263 (10)	1817 (15)	<.001	253 (10)	307 (12)	.02
Hepatomegaly and splenomegaly, not elsewhere classified	74 (3)	755 (6)	<.001	72 (3)	119 (5)	.001
Altered bowel	46 (2)	322 (3)	.007	43 (2)	67 (3)	.02
Liver disorders in diseases classified elsewhere	<10 (<1)	161 (1)	<.001	<10 (<1)	18 (<1)	.13
Neurologic						
Other and unspecified polyneuropathies	436 (17)	2483 (21)	<.001	421 (17)	526 (21)	<.001
Disorders of autonomic nervous system	56 (2)	438 (4)	<.001	53 (2)	89 (4)	.002
Male erectile dysfunction, unspecified	325 (12)	793 (7)	<.001	309 (12)	281 (11)	.22
Hereditary and idiopathic neuropathy	223 (9)	970 (8)	.39	213 (10)	236 (9)	.26
Neuralgia	94 (4)	525 (4)	.08	91 (4)	106 (4)	.28
Multisystemic						
Dyspnea	1512 (58)	5615 (46)	<.001	1460 (58)	1212 (48)	<.001
Malaise and fatigue	787 (30)	4575 (38)	<.001	757 (30)	988 (40)	<.001
Hypotension	460 (18)	2872 (24)	<.001	438 (18)	617 (25)	<.001
Syncope and collapse	364 (14)	1953 (16)	.005	338 (14)	476 (19)	<.001
Weight loss	206 (8)	1149 (10)	.009	191 (8)	298 (12)	<.001
Dizziness and giddiness	617 (24)	2852 (24)	.99	588 (24)	693 (28)	.001
Edema	828 (32)	3858 (32)	.82	788 (32)	853 (34)	.05
Pleural effusion	543 (21)	2218 (18)	.004	512 (21)	492 (20)	.79
Carpal tunnel syndrome	729 (28)	1341 (11)	<.001	708 (28)	303 (12)	<.001
Spinal stenosis, lumbar region	512 (20)	1491 (12)	<.001	493 (20)	424 (17)	.01
Glaucoma	258 (10)	1062 (9)	.08	246 (10)	316 (13)	.002
Injury of muscle, fascia, and tendon of other parts of biceps	52 (2)	80 (<1)	<.001	49 (2)	18 (<1)	.83
Arthropathy	196 (8)	733 (6)	.006	190 (8)	164 (7)	.15
Macroglossia	<10 (<1)	173 (1)	<.001	<10 (<1)	32 (1)	<.001
Nail dystrophy	70 (3)	304 (3)	.63	64 (3)	81 (3)	.15
Spontaneous ecchymoses	37 (1)	238 (2)	.06	37 (2)	48 (2)	.23
Other specified disorders of muscle	33 (1)	212 (2)	.08	32 (1)	26 (1)	.43
Spontaneous rupture of other tendons, unspecified upper arm	11 (<1)	<10 (<1)	NE^b^	11 (<1)	<10 (<1)	NE
Disturbances of skin sensation	509 (20)	2310 (19)	.67	494 (20)	497 (20)	.92

a Propensity score matching was performed on the following characteristics: age; sex: male; ethnicity: Hispanic or Latino; race: White, Black or African American; type 2 diabetes mellitus; hypertensive disease.

b NE: not evaluable.

### Ethical Considerations

This project does not meet the federal definition of human subjects’ research thus did not require institutional review board oversight or approval.

## Results

**Figure 1.**
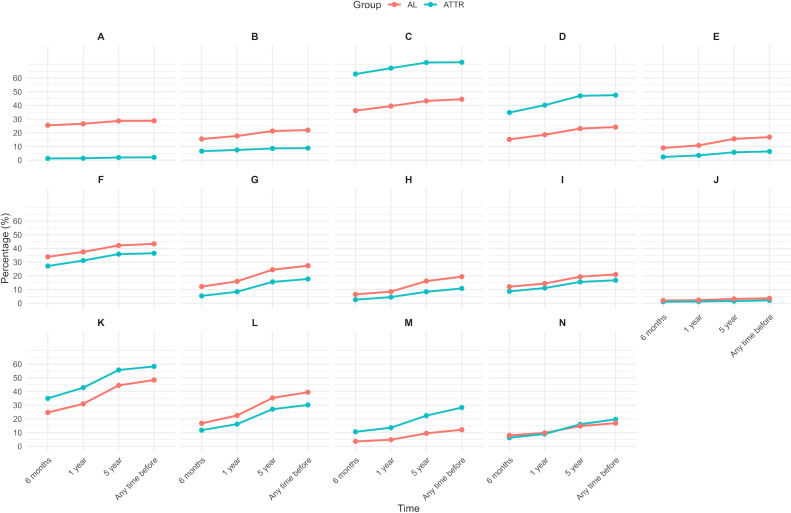
Prevalence of red-flag clinical diagnoses [1] over time prior to diagnosis of light chain amyloidosis (AL) versus wild-type transthyretin amyloidosis (ATTRwt). (A) multiple myeloma, (B) monoclonal gammopathy of undetermined significance, (C) heart failure, (D) cardiomyopathy, (E) proteinuria, (F) chronic kidney disease, (G) constipation, (H) diarrhea, unspecified, (I) other polyneuropathies, (J) disease of autonomic system, (K) dyspnea, (L) malaise and fatigue, (M) carpal tunnel syndrome, (N) spinal stenosis, lumbar region. The figure shows the two diagnoses with the greatest absolute difference in prevalence between AL and ATTR within each organ system category, assessed at any time prior to amyloid diagnosis.

We identified 12,090 patients with AL amyloidosis and 2614 with ATTRwt (Table S1 in Multimedia Appendix 1). Patients with AL were younger (mean age 68, SD 11.8 versus 77, SD 8.1 years; *P*<.001), while the ATTRwt cohort had a higher proportion of males (2149/2614, 82% vs 6355/12,090, 53%; *z*=2.85; *P*<.001). After propensity score matching by age, sex, race, and type 2 diabetes mellitus (Figure 1, Table 1), clonal plasma cell disorders were more common in AL: monoclonal gammopathy of undetermined significance (550/2503, 22% vs 220/2503, 9%; *z*=−12.93; *P*<.001) and smoldering multiple myeloma (720/2503, 29% vs 53/2503, 2%; *z*=−26.09, *P*<.001). Cardiac red-flag diagnoses were more common among patients with ATTRwt. Heart failure was present in 72% (1795/2503) of patients with ATTRwt versus 45% (1114/2503) of patients with AL (*z*=19.51; *P*<.001). Atrial fibrillation followed a similar pattern (1388/2503, 56% vs 920/2503, 37%; *z*=13.27; *P*<.001).

Renal involvement was higher in AL. Chronic kidney disease was observed in 43% (1087/2503) of patients with AL versus 37% (914/2503) of patients with ATTRwt any time prior (*z*=−4.99, *P*<.001). Proteinuria (422/2503, 17% vs 161/2503, 6%; *z*=−11.50) and nephrotic syndrome (141/2503, 6% vs <10/2503, 1%; z=−10.94) were more common in AL (*P*<.001). Gastrointestinal symptoms were more frequent in AL, including dysphagia (460/2503, 18% vs 258/2503, 10%; *z*=−8.15), constipation (27%; n=687/2503 vs 18%; n=444/2503; *z*=−8.21), nausea/vomiting (508/2503, 20% vs 304/2503, 12%; *z*=−7.82), and diarrhea (485/2503, 19% vs 270/2503, 11%; *z*=−8.49; *P*<.001 for all; Figure 1). Neurologic red flags like unspecified polyneuropathies were also higher in AL (526/2503, 21% vs 421/2503, 17%; *z*=−3.79; *P*<.001). Musculoskeletal conditions were uncommon; arthropathy was slightly more common in ATTRwt but not significantly different. Spinal stenosis was higher in ATTRwt overall (493/2503, 20% vs 424/2503, 17%; *z*=2.52; *P*=.01). Various multisystemic manifestations were more frequent in AL, such as edema (853/2503, 34% AL vs 788/2503, 32%; *z*=−1.96) and dizziness/giddiness (693/2503, 28% vs 588/2503, 24%; *z*=−3.40; *P*=.001 for both); however, dyspnea was more common in ATTRwt (1460/2503, 58% vs 1212/2503, 48%; *z*=7.03; *P*<.001). General symptoms like malaise and fatigue were more common in patients with AL (988/2503, 40% and 757/2503, 30%; *z*=−6.85; *P*<.001). Finally, carpal tunnel syndrome was more prevalent in ATTRwt (708/2503, 28% vs 303/2503, 12%; *z*=14.26; *P*<.001). Overall patterns remained consistent before and after matching, with a few exceptions that were significant prior to matching but that lost significance upon matching (male erectile dysfunction – unspecified, pleural effusion, liver disorders, injury of muscle); in addition, two diagnoses became significant after matching (dizziness and glaucoma). Findings remained consistent at 6 months, 1 year, and 5 years prior to amyloidosis diagnosis (Table S2 in Multimedia Appendix 1).

## Discussion

Our findings highlight distinct clinical patterns of diagnoses prior to a diagnosis of AL and ATTRwt. Patients with AL commonly presented with clonal diseases and renal, gastrointestinal, and neurologic manifestations, while those with ATTRwt predominantly presented with cardiac precursors like heart failure, atrial fibrillation, dyspnea, and carpal tunnel syndrome, suggesting these as key differences [1,7,9]. Despite diagnostic delays, many symptoms were identified and documented in EHRs prior to diagnosis, suggesting the potential for further work in using these data toward identifying patients with amyloidosis sooner. While our findings and prior research support this approach in both AL and ATTR [2,7,10], further work is needed to study whether EHR alerts can reduce diagnostic timelines, especially when compared to appropriate controls without amyloidosis. Strengths of our study include a large, diverse dataset and the use of propensity score matching to adjust for baseline differences. Our descriptive analysis is limited by a reliance on *ICD* codes alone, which may miss subclinical manifestations and depends on health care recognition of red-flag diagnoses. Additionally, the sensitivity and specificity of *ICD* codes for AL and ATTRwt are unknown. For example, 10% of those with ATTRwt had prior monoclonal gammopathy of undetermined significance recognition, when the actual prevalence of monoclonal gammopathy of undetermined significance in patients with ATTRwt is much higher. Additionally, given the need to include treatment for AL and ATTRwt after diagnosis, we likely missed a proportion of cases—for example, those with AL who were too sick to get chemotherapy and died before treatment initiation, and noncardiac ATTRwt where tafamidis is not approved for use. We treated all red flags equally, though some are likely more clinically indicative of amyloidosis than others. Next steps include integrating diagnostic patterns with laboratory tests, imaging, and clinical notes to develop EHR-based diagnostic tools and define time intervals between red-flag presentation and amyloidosis diagnosis.

## Supplementary material

10.2196/85803 Multimedia Appendix 1 Detailed methods.

## Abbreviations

AL: light chain amyloidosis
ATTRwt: wild-type transthyretin amyloidosis
EHR: electronic health record
ICD-10-CM: *International Classification of Diseases, 10th Revision, Clinical Modification*
