# Supplementary material for: Pre-Amyloidosis Red-Flag Clinical Diagnoses in Light Chain (AL) Versus Age-Related Transthyretin (ATTRwt) Amyloidosis: Electronic Health Record–Based Descriptive Study
Source: JMIR Med Inform. 2026 Jun 22;14:e85803. doi: 10.2196/85803 (PMC13286071; doi:10.2196/85803)
Supplement: Multimedia Appendix 1 [file medinform-v14-e85803-s001.docx]

**Supplemental Material:**

**Methods:**

We used the following plasma cell-directed therapies used to ascertain AL diagnoses: melphalan, pomalidomide, dexamethasone, bendamustine, carfilzomib, rituximab, lenalidomide, elotuzumab, ixazomib, isatuximab, venetoclax, cyclophosphamide, daratumumab, or bortezomib.

We compared the clinical characteristics, and red-flag diagnoses, of AL and ATTRwt amyloidosis after conducting a 1:1 propensity score matching on age, race, sex, and type 2 diabetes. Matching was performed using a greedy nearest-neighbor approach with a caliper of 0.1 pooled standard deviations.

The prevalence of red-flag diagnoses was evaluated at multiple time points prior to diagnosis (6 months, 12 months, 3 years, 5 years, and at any time prior). Continuous variables were compared using t-tests and categorical variables were compared using z-tests. To protect patient confidentiality, TriNetX rounds all cell counts to the nearest 10 when counts are fewer than 10, and caps age at 90 years. The following analysis reflects data extracted on June 6, 2025.

**Supplemental Table 1**. Comparison of clinical characteristics of ATTRwt or AL amyloidosis used in propensity score matching.

|  | **Before PS Matching** | | | **PS -Matched^a^** | | |
| --- | --- | --- | --- | --- | --- | --- |
|  | **ATTR** | **AL-Amyloidosis** | ***P*** | **ATTR** | **AL-Amyloidosis** | ***P*** |
|  | 2,614 | 12,090 |  | 2,503 | 2,503 |  |
| Characteristic |  |  |  |  |  |  |
| Age at Index (Mean ± SD) | 77.7 +/- 8.1 | 68.0 +/- 11.8 | <.001 | 77.3 +/- 7.9 | 77.2 +/- 7.9 | .877 |
| Sex |  |  |  |  |  |  |
| Female | 379 (15) | 5316 (44) | <.001 | 379 (15) | 394 (16) | .644 |
| Male | 2149 (82) | 6355 (53) | <.001 | 2038 (81) | 2052 (82) | .609 |
| Race |  |  |  |  |  |  |
| White | 1799 (69) | 7969 (66) | .004 | 1720 (69) | 1692 (68) | .396 |
| Black or African American | 509 (20) | 2202 (18) | .038 | 482 (19) | 513 (22) | .272 |
| Asian | 60 (2) | 492 (4) | <.001 | 57 (2) | 86 (3) | .014 |
| Other race | 44 (2) | 321 (3) | .068 | 43 (2) | 46 (2) | .748 |
| Unknown race | 179 (7) | 1003 (8) | .037 | 178 (7) | 151 (6) | .044 |
| Ethnicity |  |  |  |  |  |  |
| Not Hispanic or Latino | 2,193 | 9,416 | <.001 | 2092 (84) | 2035 (81) | .034 |
| Hispanic or Latino | 49 (2) | 570 (5) | <.001 | 48 (2) | 63 (3) | .15 |
| Unknown Ethnicity | 372 (14) | 2104 (18) | <.001 | 363 (15) | 405 (16) | .1 |
| I10-I1A Hypertensive diseases | 1875 (72) | 8557 (71) | .331 | 1811 (72) | 1873 (75) | .05 |
| E11 Type 2 diabetes mellitus | 666 (26) | 3497 (29) | <.001 | 652 (26) | 699 (28) | .135 |
| ^a^ Propensity Score matching was performed on the following characteristics: Age, Sex-Male, Ethnicity- Hispanic or Latino, Race-White, Black or African American, Type 2 diabetes mellitus, hypertensive disease | | | | | | |

**Supplemental Table 2**. Comparison of red-flag diagnoses at various timepoints prior to the diagnosis of ATTRwt or AL amyloidosis.

|  | **6 months prior dx** | | | **1-year prior dx** | | | **5-year prior dx** | | |
| --- | --- | --- | --- | --- | --- | --- | --- | --- | --- |
|  | **PS -Matched^a^** | | | **PS -Matched^a^** | | | **PS -Matched^a^** | | |
|  | **ATTR** | **AL** | ***P*** | **ATTR** | **AL** | ***P*** | **ATTR** | **AL** | ***P*** |
|  | 2,501 | 2,501 |  | 2,509 | 2,509 |  | 2,501 | 2,501 |  |
| Precursor diagnoses |  |  |  |  |  |  |  |  |  |
| Clonal |  |  |  |  |  |  |  |  |  |
| C90.0 Multiple myeloma | 32 (1) | 639 (26) | <.001 | 36 (1) | 667 (27) | <.001 | 49 (2) | 717 (29) | <.001 |
| D47.2 MGUS | 165 (7) | 388 (16) | <.001 | 188 (8) | 443 (18) | <.001 | 216 (9) | 532 (21) | <.001 |
| Cardiac |  |  |  |  |  |  |  |  |  |
| I50 Heart failure | 1574 (63) | 905 (36) | <.001 | 1687 (67) | 990 (40) | <.001 | 1782 (71) | 1083 (43) | <.001 |
| I42 Cardiomyopathy | 870 (35) | 394 (16) | <.001 | 1008 (40) | 467 (19) | <.001 | 1175 (47) | 577 (23) | <.001 |
| I48 Atrial fibrillation and flutter | 1190 (48) | 772 (31) | <.001 | 1281 (51) | 832 (33) | <.001 | 1368 (55) | 897 (36) | <.001 |
| I51.7 Cardiomegaly | 682 (27) | 381 (15) | <.001 | 858 (34) | 488 (19) | <.001 | 1102 (44) | 708 (28) | <.001 |
| I49 Other cardiac arrhythmias | 523 (22) | 408 (16) | <.001 | 666 (27) | 515 (22) | <.001 | 897 (36) | 782 (31) | .001 |
| Renal |  |  |  |  |  |  |  |  |  |
| R80 Proteinuria | 61 (2) | 225 (9) | <.001 | 87  (4) | 272 (12) | <.001 | 144  (6) | 391 (16) | <.001 |
| N18 Chronic kidney disease (CKD) | 680 (27) | 847 (34) | <.001 | 784 (31) | 940 (38) | <.001 | 898 (36) | 1056 (42) | <.001 |
| N04 Nephrotic syndrome | <10 (<1) | 89 (4) | <.001 | <10 (<1) | 97 (4) | <.001 | <10 (<1) | 128  (5) | <.001 |
| Gastrointestinal/Hepatic |  |  |  |  |  |  |  |  |  |
| K59.0 Constipation | 134 (5) | 306 (12) | <.001 | 211 (8) | 401 (16) | <.001 | 389 (16) | 613 (25) | <.001 |
| R19.7 Diarrhea | 66 (3) | 163  (7) | <.001 | 112 (5) | 213  (9) | <.001 | 209 (8) | 404 (16) | <.001 |
| R11 Nausea and/or vomiting | 83 (3) | 173  (7) | <.001 | 121 (5) | 1595 (13) | <.001 | 233 (93) | 433 (17) | <.001 |
| R13.1 Dysphagia | 75 (3) | 224  (9) | <.001 | 110 (4) | 237  (9) | <.001 | 210 (8) | 409 (16) | <.001 |
| R10 Abdominal pain | 145 (6) | 259 (10) | <.001 | 226 (9) | 358 (14) | <.001 | 480 (19) | 649 (26) | <.001 |
| K76 Other diseases of liver | 99 (4) | 116  (5) | .236 | 126 (5) | 159  (6) | .044 | 212 (9) | 246  (20) | .096 |
| R16 Hepatomegaly and/or splenomegaly | 26 (1) | 39 (2) | .105 | 35 (1) | 56 (2) | .026 | 58 (2) | 99 (4) | .001 |
| R19.4 Altered Bowels | <10 (<1) | <10  (<1) | NE | <10 (<1) | 12  (<1) | .669 | 29 (1) | 47 (2) | .037 |
| K77 Liver disorders in diseases classified elsewhere | <10 (<1) | 16  (<1) | .238 | <10 (<1) | 14  (<1) | .413 | <10 (<1) | 18 (<1) | .129 |
| Neurologic |  |  |  |  |  |  |  |  |  |
| G62 Other polyneuropathies | 218 (9) | 302 (12) | <.001 | 278 (11) | 362 (14) | <.001 | 390 (2) | 486 (19) | <.001 |
| G90 Disorders of autonomic nervous system | 28 (1) | 51  (2) | .009 | 32 (1) | 58 (2) | .006 | 41 (2) | 81 (3) | <.001 |
| N52.9 Male erectile dysfunction | 70 (3) | 62  (3) | .48 | 100 (4) | 102 (4) | .886 | 214 (9) | 203  (8) | .574 |
| G60 Hereditary and idiopathic neuropathy | 67 (3) | 74 (3) | .55 | 95 (4) | 98 (4) | .826 | 170 (7) | 186  (7) | .379 |
| M79.2 Neuralgia | 22 (<1) | 25 (1) | .66 | 26 (1) | 29 (1) | .684 | 60 (2) | 68 (3) | .474 |
| Multisystemic |  |  |  |  |  |  |  |  |  |
| R06.0 Dyspnea | 876 (35) | 618 (25) | <.001 | 1074 (43) | 777 (31) | <.001 | 1393 (56) | 1113 (45) | <.001 |
| R53 Malaise and fatigue | 296 (12) | 420 (17) | <.001 | 407 (16) | 564 (23) | <.001 | 678 (27) | 883 (35) | <.001 |
| I95 Hypotension | 204 (8) | 311 (12) | <.001 | 263 (11) | 375 (15) | <.001 | 399 (16) | 549 (22) | <.001 |
| R55 Syncope | 127 (5) | 172 (7) | .007 | 168 (7) | 232 (9) | .001 | 290 (11) | 1661 (14) | <.001 |
| R63.4 Weight loss | 58 (2) | 92  (4) | .005 | 91  (4) | 127  (5) | .013 | 165 (7) | 251 (10) | <.001 |
| R42 Dizziness | 157 (6) | 199 (8) | .021 | 235 (9) | 286 (11) | .018 | 473 (19) | 570 (23) | .001 |
| R60 Edema | 325 (13) | 405 (16) | .001 | 454 (18) | 517 (21) | .024 | 718 (29) | 783 (31) | .045 |
| J90 Pleural effusion | 270 (11) | 267 (11) | .891 | 345 (14) | 348 (14) | .902 | 491 (20) | 470 (19) | .451 |
| Other |  |  |  |  |  |  |  |  |  |
| G56.0 Carpal tunnel syndrome | 265 (11) | 90  (4) | <.001 | 342 (14) | 120 (5) | <.001 | 559 (22) | 238  (10) | <.001 |
| M48.06 Spinal stenosis, lumbar region | 158 (6) | 199  (8) | .024 | 225 (9) | 245  (10) | .333 | 403 (16) | 371 (15) | .211 |
| H40-H42 Glaucoma | 122 (5) | 177  (7) | .001 | 145 (6) | 211  (8) | <.001 | 219 (9) | 282 (11) | .003 |
| S46.2 Injury of muscle, fascia and tendon of other parts of biceps | <10 (<1) | <10 (<1) | NE | 12 (<1) | <10  (<1) | NE | 37 (2) | 17 (<1) | .006 |
| M12.9 Arthropathy | <10 (<1) | 16 (<1) | .238 | 15 (<1) | 23  (<1) | .193 | 85 (3) | 79 (3) | .634 |
| K14.8 Macroglossia | <10 (<1) | 12  (<1) | .669 | <10 (<1) | 15  (<1) | .316 | <10 (<1) | 27 (1) | .005 |
| L60.3 Nail dystrophy | 12 (<1) | 77 (1) | .288 | 18 (<1) | 30  (1) | .082 | 44  (2) | 63  (3) | .063 |
| R23.3 Spontaneous ecchymosis | <10 (<1) | 14  (<1) | .413 | 13 (<1) | 20  (<1) | .221 | 29  (1) | 41  (2) | .149 |
| M62.89 Other disorders of muscle | <10 (<1) | <10  (<1) | NE | 11 (<1) | <10  (<1) | NE | 21 (<1) | 20 (<1) | .875 |
| M66.829 Spontaneous rupture of other tendons | 0 (0) | 0 (0) | NE | 0 (0) | 0 (0) | NE | 0 (0) | 0 (0) | NE |
| R20 Disturbances of skin | 100 (4) | 106 (4) | .669 | 148 (6) | 155  (6) | .678 | 371 (15) | 389 (16) | .478 |
| ^a^ Propensity Score matching was performed on the following characteristics: Age, Sex-Male, Ethnicity- Hispanic or Latino, Race-White, Black or African American, Type 2 diabetes mellitus, hypertensive disease | | | | | | | | | |
